# *Blomia tropicalis* Blo t 5 and Blo t 21 recombinant allergens might confer higher specificity to serodiagnostic assays than whole mite extract

**DOI:** 10.1186/1471-2172-14-11

**Published:** 2013-02-27

**Authors:** Kellyanne dos Anjos Carvalho, Osvaldo Pompílio de Melo-Neto, Franklin Barbalho Magalhães, João Carlos Marques Ponte, Filipe Adriano Borba Felipe, Mariese Conceição Alves dos Santos, Givaneide dos Santos Lima, Álvaro Augusto Cruz, Carina Silva Pinheiro, Lain Carlos Pontes-de-Carvalho, Neuza Maria Alcantara-Neves

**Affiliations:** 1 Laboratório de Alergia e Acarologia, Instituto de Ciências da Saúde, Universidade Federal da Bahia, Avenida Reitor Miguel Calmon, sem nº, Canela, Salvador, Bahia, CEP 40110-100, Brazil; 2 Centro de Pesquisas Aggeu Magalhães, Fundação Oswaldo Cruz, Recife, Pernambuco, Brazil; 3 Associação Caruaruense de Ensino Superior, Caruaru, Pernambuco, Brazil; 4 Instituto de Matemática, Universidade Federal da Bahia, Salvador, Bahia, Brazil; 5 Centro de Estudos de Alergias Respiratórias, Salvador, Bahia, Brazil; 6 ProAR – Nucleo de Excelência em Asma, Universidade Federal da Bahia, Salvador, Bahia, Brazil; 7 Centro de Pesquisas Gonçalo Moniz, Fundação Oswaldo Cruz, Salvador, Bahia, Brazil

**Keywords:** *Blomia tropicalis*, Recombinant allergens, Immunodiagnosis, Cross-reactivity, *Ascaris lumbricoides*, Sensitivity, Specificity

## Abstract

**Background:**

*Blomia tropicalis* is a dust mite and an important source of allergens in tropical regions. Up to now, the assays to diagnose atopy to this mite use whole body extract as antigens. However, anti-*B. tropicalis* IgE antibodies cross-react with *Ascaris lumbricoides* antigens, hindering the diagnosis of allergy to this mite. In this study, *B. tropicalis* recombinant allergens were evaluated with the purpose of developing an immunodiagnostic assay for allergy to this mite with greater specificity than those commercially available.

**Methods:**

Two *B. tropicalis* allergens (Blo t 5 and Blo t 21) were cloned into a plasmidial expression vector, expressed in *Escherichia coli* and purified by affinity chromatography. Sixty-three sera containing anti-*B. tropicalis* extract (BtE) IgE antibodies were used to investigate IgE reactivity to the recombinant Blot 5 and 21 allergens. Inhibition assays with 20 sera pre-adsorbed with *A. lumbricoides* extract were performed using rBlo t 5, rBlo t 21, and BtE as antigens. All the assays were carried using indirect ELISA.

**Results:**

Eighty-two point nine percent and 80.0% of the sera with anti-BtE antibodies from 35 children reacted with rBlo t 5 and rBlo t 21, respectively, whereas 92.8% and 89.3% of the 28 sera with anti-BtE antibodies from adult asthma patients reacted with the same allergens, and 96.4% of these sera reacted with a mixture of rBlo t 5 and rBlo t 21. In an inhibition ELISA, the absorption of sera by *A. lumbricoides* extract affected less the reaction with rBlo t 5 and rBlo t 21 than with BtE*.*

**Conclusions:**

The rBlo t 5 and rBlo t 21 allergens contain important epitopes recognized by IgE antibodies of individuals allergic to *B. tropicalis* antigens. Moreover, the assays using the recombinant allergens had lower IgE cross-reactivity with *A. lumbricoides* antigens, a fact which would confers higher specificity to serodiagnostic assays than the crude mite extract. However, additional recombinant allergens should be evaluated in order to reach the same sensitivity of the commercially available assays based on mite extract.

## Background

Allergic respiratory diseases, like asthma and rhinitis, are worldwide spread and their prevalences have increased over the past decades, reaching epidemic proportions [1]. Mite-allergen sensitization is a well-documented risk factor for asthma and allergic diseases in atopic individuals. In tropical regions of the world, *Blomia tropicalis* and *Dermatophagoides pteronyssinus* are predominant in house dust and are commonly found together [2,3]. *B. tropicalis* mite is the major source of indoor allergens in the northeastern cities of Brazil [4,5]. Clinical history, skin prick test (SPT) results and specific IgE detection are the tripod for the diagnosis of allergic diseases. The majority of the methods for detecting anti-allergen IgE antibodies is based on the use of natural allergenic extracts, a fact that may compromise the interpretation of their results. Natural allergen extracts are highly complex molecular mixtures, containing many proteins, of which only some have allergenic properties, and with their relative contents varying greatly from one extract to another [6,7]. Caraballo and collaborators [2] described at least 25 IgE-binding protein bands in *B. tropicalis* crude extract. Around 12 recombinant allergens have been molecularly and immunologically characterized and deposited into the allergen database, according to International Union of Immunological Societies (IUIS) Allergen Nomenclature (http://www.allergen.org). Among these allergens, it includes Blo t 1 (cysteine protease, 26 kDa), Blo t 3 (trypsin protease, 25 kDa), Blo t 5 (unknown molecular function, 14 kDa), Blo t 11 (paramyosin, 110 kDa), Blo t 12 (unknown molecular function, 14 kDa), and Blo t 21 (unknown molecular function, 13.2 kDa), which are present in 50% or more of the sera from *B. tropicalis*-sensitized individuals [3,4]. Blo t 5 and Blo t 21 represent the major allergens in *B. tropicalis* mite. These allergens share some structural similarities determined by NMR consisting of three anti-parallel α-helices, assembled in a helical bundle. Despite the fact that some putative IgE epitope residues are conserved in both Blo t 5 and Blo t 21 three-dimensional structures, these allergens present a low to moderate cross-reactivity [5,8].

Nevertheless, the IgE-binding properties to these allergens may vary depending on several factors, such as genetic background of the study population, frequency of mite sensitization, and intrinsic molecular characteristics of the allergens, such as isoform expression profile [3]. Furthermore, in tropical regions of the world, where the high prevalence of helminth infections is a public health problem, the serodiagnosis of allergic diseases may be compromised by cross-reactive antibody to epitopes shared by mite and helminths [9,10].

In this study we produced *B. tropicalis* recombinant allergens (rBlo t 5 and rBlo t 21) in a prokaryotic system, based on sequences obtained in the GenBank database. A total of 63 sera with IgE antibodies reacting with *B. tropicalis* extract (BtE), from asthmatic patients and from atopic children from Salvador, a large urban center in northeastern Brazil, were used to investigate IgE reactivities to the recombinant Blo t 5 and Blo t 21 allergens by indirect ELISA. The cross-reactivities of IgE antibodies between *B. tropicalis* (crude extract and recombinant allergens) and *A. lumbricoides* (crude extract) were also evaluated.

## Methods

### *B. tropicalis* and *A. lumbricoides* extracts

An ether-treated extract of *B. tropicalis* mite, obtained from house dust samples in Salvador, was prepared according to Baqueiro and collaborators [11]. Briefly, *B. tropicalis* was collected from bed dust in Salvador, Brazil, cultured with a fish food medium, at 25°C and 75% humidity and cloned. The mites were purified from the medium by flotation on a 5 M sodium chloride solution, followed by several washings by filtration in a 100 μm-pore polystyrene sieve with endotoxin-free distilled water. The washings were carried out until no food residues were seen under microscopy. The mites were lysed in 0.15 M phosphate-buffered saline, pH 7.4 (PBS), in an electric blender (Waring Commercial, Torrington, CN, USA). Lipids from the lysate were extracted by five or six ether extractions and discarded. The protein content of the *B. tropicalis* aqueous extract was determined by the Folin reagent method [12]. The extract was standardized using a commercially available capture ELISA for detection of Blo t 5 allergen. (INDOOR Biotechnologies, Charlottesville, VI, USA), with the Blo t 5 content in the extract established at 200 ng per mg of total protein, and subsequently aliquoted and cryopreserved until use. The *A. lumbricoides* aqueous extract was prepared from adult worms obtained from infected albendazol and bisacodyl-treated children in Salvador, Brazil, according to Ponte and collaborators [9].

### *B. tropicalis* recombinant allergens

Sequences encoding *B. tropicalis* allergens (Blo t 5 and Blo t 21) were obtained from their deposits in the GenBank database. The selection criterion was based on published analysis of the frequency of allergic patients' sera with IgE antibody reactivity to the recombinant allergens (50% or more reactivity with the tested sera) [3,4]. Pairs of primers (5^′^-3^′^) were designed based on the published full length nucleotide sequence of Blo t 5 and Blo t 21 (GenBank: U59102 and GenBank: AY800348, respectively), which allowed their amplification by polymerase chain reaction (PCR) flanked by restriction enzyme sites for BamHI/XhoI (Blo t 5, forward – CCC**GGATCC**ATGAAGTTCGCCATCGTTC; reverse - GGG**CTCGAG**TTATTGGGTTTGAATATC) or BamHI/HindIII (Blo t 21, forward - CCC**GGATCC**ATGAAATTTATCATCGCATTG; reverse – GGG**AAGCTT**CTATTCGGAATCTTGGAC). As a *B. tropicalis* cDNA library had already been constructed in our laboratory, an aliquot of it was used as template for the PCR. The Blo t 5 and Blo t 21 amplicons were ligated to the pRSETA expression vector and used for transforming DH-10 B-strain *Escherichia coli*. The bacteria were cultivated and plasmid DNAs were obtained and sequenced. All sequences were aligned and homology analysis was performed using the BLAST network service provided by NCBI. Constructs containing Blo t 5 and Blo t 21 were then used to transform BL21(DE3) *E. coli* BL21(DE3)pLysS, generating His (histidine)-tagged proteins (His-rBlo t 5 and His-rBlo t 21) after induction with 0.1 mM isopropyl β-D-thiogalatoside (IPTG). The recombinant proteins were subsequently purified by affinity chromatography using the Ni-NTA agarose resin (QIAGEN Biotecnologia Brasil Ltda; São Paulo, SP, Brazil), according to the manufacturer's protocol.

### Polyacrylamide gel electrophoresis and IgE immunoblot assays

His-rBlo t 5 and His-rBlo t 21 recombinant proteins were subjected to polyacrylamide gel electrophoresis in the presence of sodium dodecyl sulphate (SDS-PAGE; Merck & Co., Inc., White House Station, NJ, USA), according to Laemmli [13], using a Mini-PROTEAN III Electrophoresis Cell (Bio-Rad Laboratories, Hercules, CA, USA), followed by electroblotting onto nitrocellulose membrane (Sigma, St Louis, MO, USA). Immunoblotting was then carried out to determine the reactivities of anti-allergen serum IgE antibodies to these fusion proteins. Briefly, the blots were blocked with 0.15 M phosphate-buffered saline, pH 7.2 (PBS) containing 0.05% Tween (PBS/T) and 10% (w/v) dry non-fat milk for at least two hours at room temperature, followed by overnight incubation with continuous shaking at 4°C with human sera diluted 1:10 in PBS/T containing 5% dry non-fat milk. The blots were incubated with mouse anti-human IgE:HRP monoclonal antibody (ABCAM,Cambridge, UK) at room temperature for one hour. The reaction was developed using H_2_O_2_ and diaminobenzidine (DAB) as substrate and chromogen, respectively (Sigma, St. Louis, MO, USA). Between all steps, the blots were washed three times with PBS/T followed by two washes with PBS alone.

### Serum samples

Thirty five of the 63 studied sera were obtained from atopic children living in Salvador, Bahia, Brazil. Atopy in these children was characterized by a positive result in the ImmunoCAP assay (Phadia Diagnostics AB, Uppsala, Sweden) for at least one of four tested allergens (from *B. tropicalis*, *D. peteronyssinus*, *Blattella germanica* and *Periplaneta americana*). The children, aged 4 to 11 years, were enrolled in the SCAALA (Social Change in Asthma and Allergy in Latin America) program, a study performed in 2005 to study risk factors for asthma and allergies in children [14]. The 28 remaining sera were from adult patients with moderate to severe asthma attending the outpatient facility of the Centro de Estudos em Alergias Respiratórias (CEAR), Salvador, Brazil. The cross-reactivity between *B. tropicalis* recombinant allergens and *A. lumbricoides* antigens was assessed in 20 adult sera from the CEAR patients. Informed consent was obtained from the SCAALA project children’s parents or guardians and ethical approval was granted by the Instituto de Saúde Coletiva of the Universidade Federal da Bahia, Salvador, Brazil, and by the National Commission on Ethics in Research (CONEP), Brazil. Written consent was obtained from all adult asthma patients and ethical approval was provided by the Ethical Committee of the Maternidade Climério de Oliveira, Universidade Federal da Bahia, Salvador, Brazil (protocol no. 044/2010).

### ELISA for human IgE antibody reactivity with *B. tropicalis* recombinant proteins

ELISA was performed in microassay plate (Costar, Bethesda, MD, USA) wells, which were incubated with 100 μg of *B. tropicalis* extract or 5 μg of each fusion proteins (His-rBlo t 5 or His-rBlo t 21) when tested individually or 2.5 μg of each when used mixed together in the same solid phase (rBlo t 5 plus rBlo t 21), per mL of sodium carbonate-bicarbonate buffer, pH 9.6, overnight at 4°C. The reaction was developed as described previously [9] and the IgE cut-off for the BtE, rBlo t 5 or rBlo t 21 assays defined according to the mean plus two standard deviations of the results obtained using sera from 10 individuals without history of allergy and with negative SPT reaction to the tested allergens [9].

### Detection of anti-*B. tropicalis* IgE antibodies cross-reactive with *A. lumbricoides* antigens

Inhibition ELISA was performed to investigate the presence of anti-*B. tropicalis* extract, anti-rBlo t 5, and anti-rBlo t 21 IgE antibodies that cross-reacted with *Ascaris lumbricoides* antigens. The inhibition was accomplished by pre-incubating the sera with 0.3 or 3 μg/mL of *A. lumbricoides* extract. These concentrations were chosen based on previous work [9] and are within the concentration range that led to the greatest differences between non-absorbed and absorbed sera. As control for the specificity of the inhibition of the binding of anti-*B. tropicalis* IgE antibodies, total IgE was determined in the same sera, before and after absorption with *Ascaris* extract. The assay was carried out as described elsewhere [9]. Results were expressed in percentage of reduction and specific sIgE levels in sera non-adsorved and adsorved with *Ascaris* extract (OD 450 nm). The percentage of reduction in anti-BtE anti-rBlo t 5 and anti-rBlo t 21 IgE antibodies or total IgE level = [1 - (mean OD obtained in duplicates of serum that had been pre-incubated with *A. lumbricoides* antigen/mean OD obtained in duplicates of untreated serum )] x 100.

### Statistical analysis

The D’Agostino and Pearson test was used for verification of the data normality. The significance of the differences among the different allergens was assessed by the Wilcoxon signed rank test (when the data distribution was found to be non-Gaussian). Differences with a P value equal to or less than 0.05 were considered statistically significant.

## Results

### Expression and validation of recombinant fusion proteins

**Figure 1:**
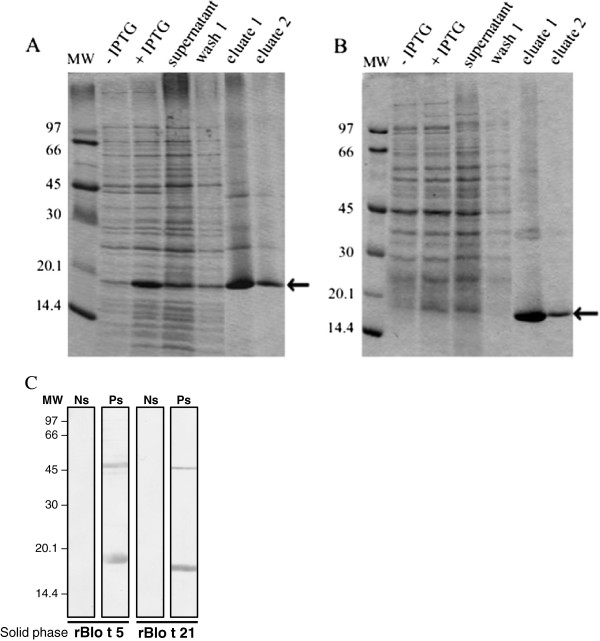
**Expression and purification of recombinant proteins rBlo t 5 and rBlo t 21.** The his-tagged proteins rBlo t 5 (**A**) and rBlo t 21 (**B**) were induced by IPTG and affinity-purified using the resin Ni-NTA agarose. The samples collected during expression induction and purification were analyzed by 15% SDS-PAGE stained with Coomassie Blue. Arrows indicate purified proteins. (**C**) Immunoblotting of the rBlo t 5 (A) and rBlo t 21 with negative control sera (Ns) and positive sera – asthmatic patient (Ps). MW, molecular weight.

*B. tropicalis* recombinant allergens were expressed and purified as full length fusion proteins containing N-terminal His-tags (6 His). Once the fusion proteins were purified, their tags (6 His) were not removed. The achieved yields of rBlo t 5 and rBlo t 21 were 3.1 to 4.6 mg and 2.9 to 4.5 mg per liter of bacterial culture, respectively. Each His-tagged (rBlo t 5 and rBlo t 21) proteins produced an expected band in SDS-PAGE (Figure 1A and B). Validation of recombinant fusion proteins was performed by IgE immunoblotting, in which antibodies from atopic and asthmatic individuals, but not negative control sera, were found to react with rBlo t 5, rBlo t 21.

### Antigenicity of *B. tropicalis* recombinant proteins

**Figure 2:**
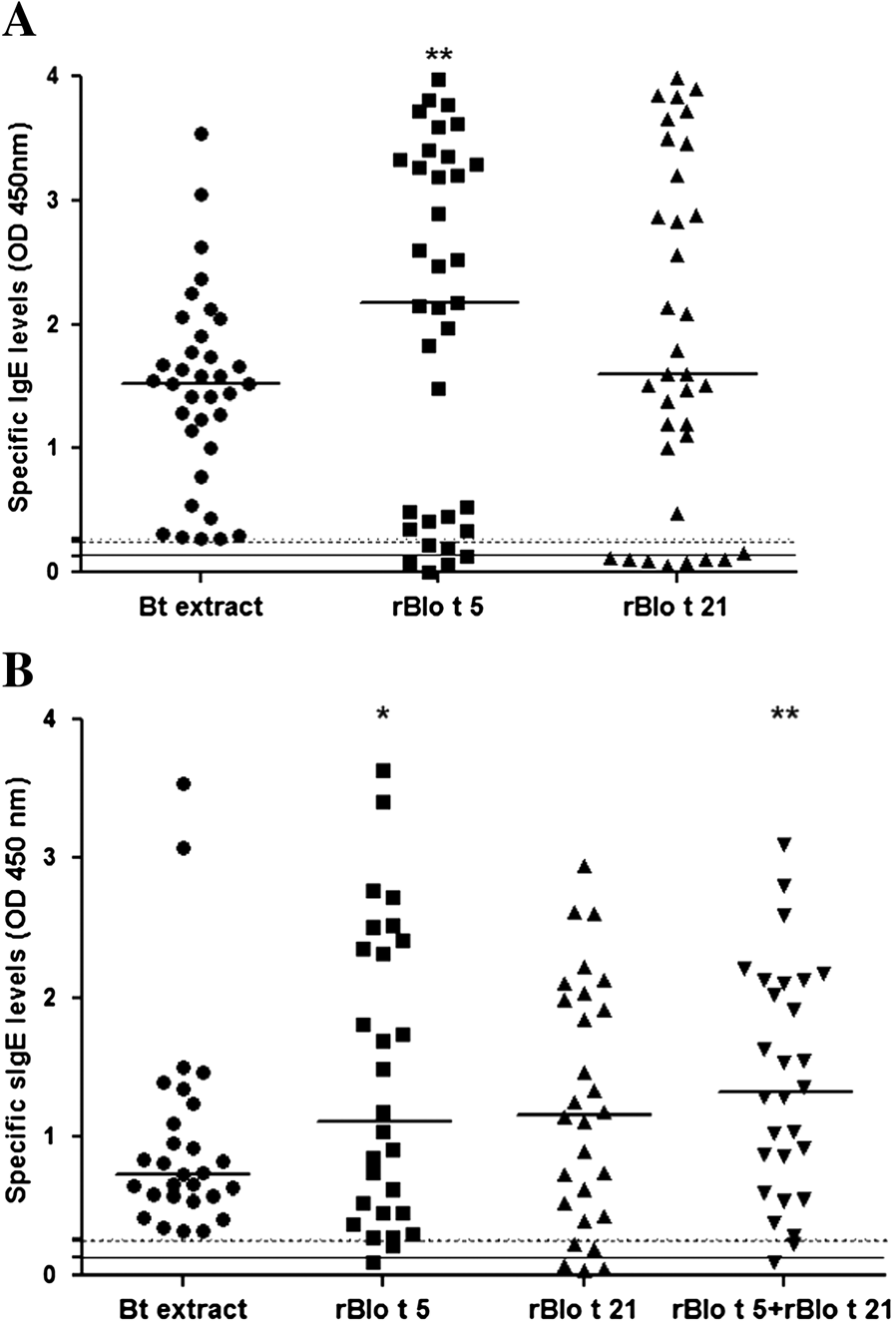
**Antigenicity of rBlo t 5 and rBlo t 21.** IgE antibody levels were assessed by means of indirect ELISA in sera of atopic children (**A**) and asthmatic adult individuals (**B**) with antibodies against whole *B. tropicalis* extract. Each symbol represents the result obtained with an individual serum. Short horizontal solid lines represent the median value of the group results. The cut-offs are represented by horizontal dotted (0.269 for BtE assay), dashed (0.250 for rBlo t 5 assay) or solid (0.136 for r Blo t 21) lines, respectively. A) ** rBlo t 5 compared with Bt extract, p < 0.0067 and B)* rBlo t 5 compared with Bt extract, p < 0.0314, ** rBlo t 5 plus rBlo t 21 compared with Bt extract, p < 0.0034, Wilcoxon signed rank test.

Indirect ELISA was used to evaluate the reactivity of IgE antibodies with the fusion His-rBlo t 5 and His-rBlo t 21 fusion proteins. The IgE reactivity profile of the tested sera with the recombinant proteins is summarized in Table 1. The panel of 35 sera from children with IgE antibodies against BtE had IgE antibody reactivity percentages of 82.9% (29 sera out of 35) for rBlo t 5 and 80% (28 out of 35) for rBlo t 21. The IgE reactivity percentages for the 28 sera from adult asthma patients with IgE antibodies against BtE were 92.8% (26 out of 28) for rBlo t 5 and 89.3% (25 out of 28) for rBlo t 21. When the indirect ELISA assay was performed using a mixture of rBlo t 5 and rBlo t 21 allergens, a positive reaction was observed in 96.4% (27 out of 28) of the sera (Table 1). Despite the fact that some sera reacted with BtE but not with the recombinant allergens, the levels (expressed by OD_450 nm_) of IgE antibodies against rBlo t 5 in the children group, and of IgE antibodies in the adult asthmatic patient group as detected in assays using rBlo t 5 or a mixture of rBlot 5 plus rBlo t 21, were significantly higher than in the assay using BtE (p < 0.0067; p < 0.0314 and p < 0.0034, Wilcoxon signed rank test; Figure 2, A and B). Although the percentages of IgE antibody reactivity to the recombinant antigens were higher in the adult patients' serum group than in the children's serum group, there were no statistically significant differences between these two populations (p > 0.05, Mann-Whitney's test, data not shown). Of the total sera panel, 66.7% (42 out of 63 children's and adults' sera) reacted with both rBlo t 5 and rBlo t 21; 92.1% of these sera (58 out of 63) reacted with at least one of the recombinant allergens and 7.9% (5 out of 63) recognized only the *B. tropicalis* crude extract (data not shown).

**Table 1:** **Reactivity of anti-*B. tropicalis *IgE antibodies, assessed by ELISA, using recombinant proteins (rBlo t 5 and rBlo t 21) and crude extract as antigens, in sera containing anti-*B. tropicalis *extract IgE antibodies**

**Antigens**	**N**^**o**^**. of sera with positive results (%)**	**N**^**o**^**. of sera with negative results (%)**
	Sera from 35 children with anti-*B. tropicalis* extract IgE antibodies	
rBlo t 5	29 (82.9)	6 (17.1)
rBlo t 21	28 (80.0)	7 (20.0)
	Sera from 28 adult asthma patients with anti-*B. tropicalis* extract IgE antibodies	
rBlo t 5	26 (92.8)	2 (7.2)
rBlo t 21	25 (89.3)	3 (10.7)
rBlo t 5 + rBlo t21	27 (96.4)	1 (3.6)

### Cross-reactivity of IgE antibodies against *B. tropicalis* extract and recombinant allergens with *A. lumbricoides* antigens

**Figure 3:**
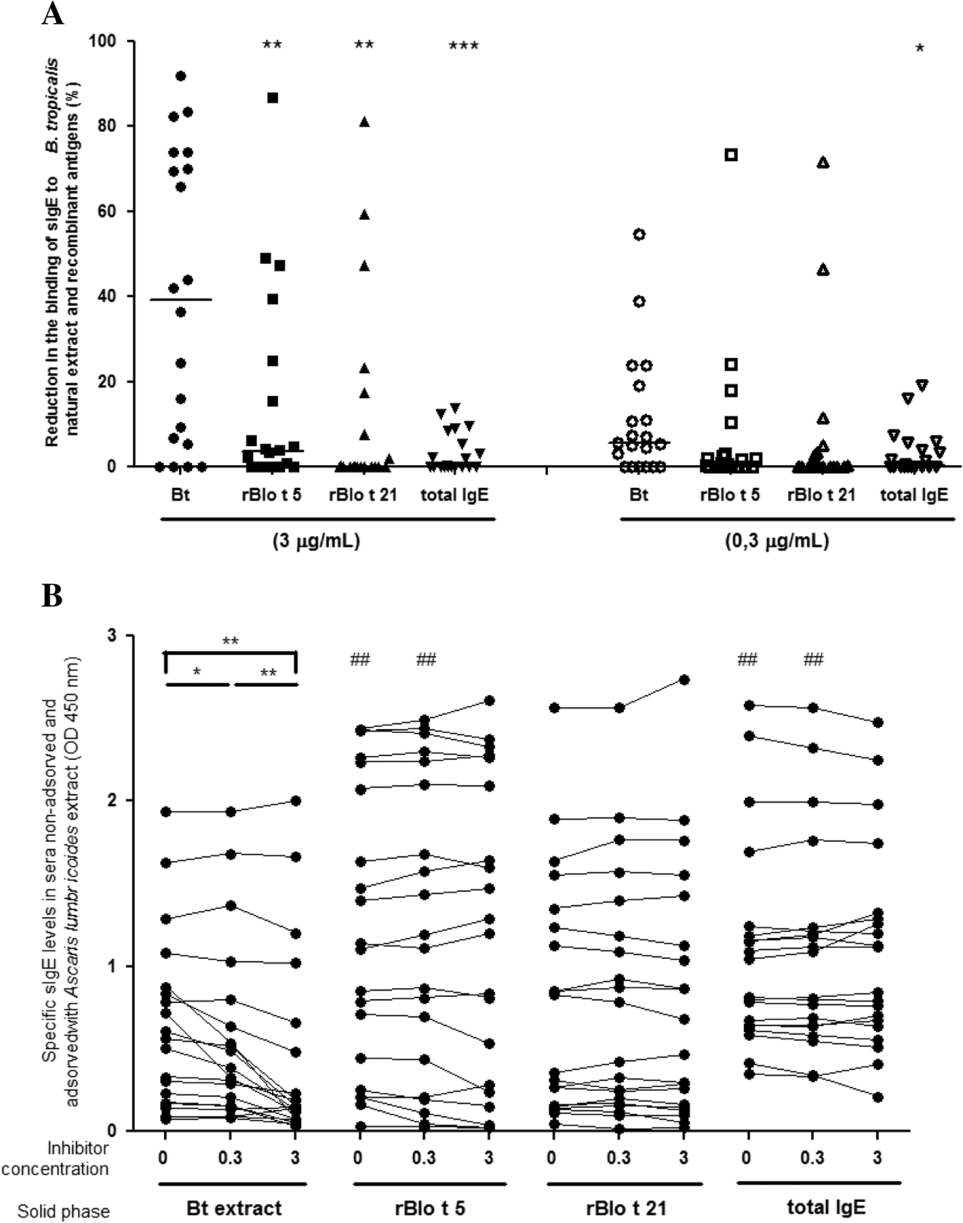
**Reaction of anti-*B. tropicalis*, recombinant allergen IgE antibodies and total IgE to *A. lumbricoides *extract.** Twenty adults' sera with anti-*B. tropicalis* extract IgE antibodies were pre-incubated with the indicated concentrations of *A. lumbricoides* crude extract and tested in indirect ELISAs using *B. tropicalis* crude extract (BtE) or recombinant allergens (rBlo t 5 and rBlo t 21) as antigens or assayed for total IgE levels. The reductions in anti-Bt, anti-rBlo t 5, and anti-rBlo t 21 IgE antibody levels or total IgE levels were calculated as described in the Methods section (**A**), * p < 0.0231, ** p < 0.0020 and *** p < 0.0008; Wilcoxon signed rank test. (**B**) Specific sIgE levels in sera non-adsorved and adsorved with *Ascaris lumbricoides* extract (OD 450 nm), * p < 0.0217 ** p < 0.0012, # p < 0.0030, ## p< 0.0041; Wilcoxon signed rank test.

There was a higher reduction in the levels of anti-*B. tropicalis* crude extract IgE antibodies (median inhibition of 39.2%) than in the levels of anti-rBlo t 5 (median inhibition of 3.6%) and anti-rBlo t 21 (median inhibition of 0.1%) IgE antibodies, when they were incubated with *A. lumbricoides* extract at both concentrations (Figure 3A). However, the difference between the recombinant protein and the BtE assays was statistically significant only with the concentration of 3 μg/mL of *A. lumbricoides* extract (p < 0.0052 for rBlo t 5 and p < 0.0020 for rBlo t 21; Wilcoxon signed rank test). As expected, the anti-*B. tropicalis* crude extract IgE antibody levels were lower in sera pre-adsorbed with *A. lumbricoides* extract than in the non-adsorbed sera (Figure 3B). This difference between the non-adsorbed and adsorbed sera was statistically significant for both concentrations, 0.3 μg/mL and 3 μg/mL of *A. lumbricoides* extract (p < 0.0217 and p < 0.0012; Wilcoxon signed rank test). Furthermore, there was also statistically significant difference between sera adsorved with 0.3 μg/mL and 3 μg/mL of *A. lumbricoides* extract (p < 0.0011; Wilcoxon signed rank test). The proportion of total IgE that was reduced by incubation with *A. lumbricoides* natural extract (median inhibition of 0.1%) was lower than the proportion of anti-*B. tropicalis* extract IgE antibodies that was reduced by the same treatment (p < 0.0008, Wilcoxon signed rank test; Figure 3).

## Discussion

The *B. tropicalis* mite has been the target of several studies highlighting its role as being one of the major asthma sensitizing agent in tropical areas of the world [15-17]. These studies have demonstrated that serum IgE antibodies in 42% to 98% of *B. tropicalis*-sensitized individuals react with Blo t 5 and Blo t 21 [2,14]. In the present work it is shown that a large proportion of serum IgE antibodies from BtE-sensitized children and adults with asthma reacted with rBlo t 5 and rBlo t 21 obtained as described herein. The rBlo t 5 in acidic pH solutions, like under purification denaturing conditions, tends to form molecular aggregates [8]. Likely, the rBlo t 21 behave like the rBlo t 5, forming aggregates under the same conditions, since both allergens share structural similarities [5]. This could explain the nonspecific band recognition by antibodies from asthmatic patient sera that were used in immunoblotting assay of this work. *B. tropicalis* is found more frequently (71.8%) than *D. pteronyssinus* (39.9%) in bed dust in Salvador, a major city in Northeast of Brazil [18]. In environments where *B. tropicalis* predominates in the mite fauna, it is common to find a high rate of co-sensitization to Blo t 5 and Blo t 21, as highlighted in our study (in 66.7% of 63 sera from *B. tropicalis* sensitized individuals) [4,19]. Although several individuals had a higher IgE antibody reactivity to rBlo t 5 and rBlo t 21 than to BtE (i.e., their sera led to higher optical densities when tested in ELISA for IgE antibodies against these recombinant allergens than against the extract; Figure 2), there is a small percentage of sera (7.9%, corresponding to 5 out of 63 sera) that does not recognize any of the recombinant allergens, a fact that would limit the sensitivity of assays using these allergens. When the two recombinant allergens were used together in the same immunoassay, the IgE reactivity increased so that 96.4% of the sera from asthmatic patients had anti-BtE antibodies. Some studies indicate that mite allergy could be detected using a mixture of two or more major recombinant allergens (component-resolved diagnostics), replacing the natural extract [20]. Furthermore, the use of multiple recombinant allergens to detect sensitization profiles could be very important to guide and improve immunotherapy for mite allergies [19-21].

This study, despite not comparing the reactivity of IgE antibodies to rBlo t 5 and rBlo t 21 with the reactivity to their native counterparts, suggests that recombinant allergens produced as described in this paper can replace natural allergen extracts in the diagnosis of allergies, confirming previously published data showing that rBlo t 5 expressed in *E. coli* and native rBlo t 5 have comparable IgE reactivity in terms of percentage of sera with antibodies [22]. However, our data also indicate that it is desirable to introduce other recombinant antigen(s), in addition to rBlo t 5 and rBlo t 21, in an assay for IgE antibodies to diagnosis hypersensitivity to *B. tropicalis* in order to increase its sensitivity. Blo t 7, which has been shown to be as reactive as Blot t 5 with IgE antibodies in the sera from allergic children in a study also carried out in a tropical environment [19], may be a good candidate to be included to a pool of recombinant antigens to increase the sensitivity of an immunoassay. Meanwhile, Blo t 10 and Blo t 11, which shown to cross-react with *A. lumbricoides*[23], would not be good candidates to be included in an immunoassay with multiple recombinant antigens.

The relationship between house dust mite sensitization and triggering of asthma and other atopic diseases is well documented [24,25]. Given the difficulty of completely eliminating the sensitizing agents of the affected individuals' homes, immunotherapy has been playing a key role in alleviating the clinical aspects of allergic diseases. Thus, a more specific mite allergy diagnosis is necessary and it is also a key point to develop appropriate, more specific and individualized therapies [26]. The antigenic extracts used in the diagnosis and immunotherapy of allergic diseases are obtained from natural sources. This fact brings many disadvantages, such as the presence of large amounts of non-antigenic proteins; contamination by other potentially immunostimulating compounds, like endotoxin; allergen variability in the sample composition (depending on the season of the year, the mite life cycle and differences in protein extraction protocols). In addition, the more sensitizing allergenic proteins in the mite extract can be found in low concentrations, requiring the use of higher doses of total extract, which is not always convenient in clinical practice [20,25,27,28].

In addition to the problems described above with the use of whole allergenic extracts in the immunodiagnosis of allergic diseases, there is another factor that should be considered by the companies that commercialize these products: the common association of *B. tropicalis* sensitization with helminth infection in tropical and undeveloped regions of the world. It is in fact believed that about 1.5 billion people worldwide are infected with *A. lumbricoides*[10,29]. Salvador city, where the donors of the sera used in the present work live, is one helminth parasite endemic areas in Brazil, having high prevalence of *A. lumbricoides*, *Trichuris trichiura* and *Toxocara* spp (*Toxocara canis* and *Toxocara cati*) infections. In fact, most of the sera studied in the present work had anti-*Ascaris* IgG antibodies, indicating present and/or past *A. lumbricoides* infection. In this area, an overlap of parasitic endemicity and high prevalence of allergic diseases is observed [30-32]. There is often dissociation between positive skin prick test results and detectable serum IgE antibodies in allergic individuals who are also co-infected with *A. lumbricoides*[9]. This finding could be explained by the existence of IgE antibodies, raised in response to the helminth infection, that cross-react with allergens but are unable to lead to degranulation of mast cells, consequently increasing anti-allergen IgE antibody levels but not SPT positivity [9,33]. The increase of knowledge on cross-reactive IgE antibodies to common environmental respiratory allergens and helminth antigens will certainly improve allergy diagnosis and perhaps even immunotherapy. In the present study, pre-incubation of sera with *A. lumbricoides* extract led to varying degrees of inhibition of the binding of IgE to mite allergens. It would also be useful to further demonstrate this cross-reaction by inhibiting the binding of IgE antibodies to *A. lumbricoides* extract by incubation of the sera with *B. tropicalis* antigens. As the high levels of IgG antibodies raised in the immune response to this helminth hinders the detection of IgE anti-*Ascaris* antibodies, a possible way to carry out the experiment would be to use an assay to detect IgE antibodies more sensitive than the one used in the present work.

The inhibition by adsorption with *A. lumbricoides* extract was higher for the binding of antibodies to *B. tropicalis* crude extract than to rBlot 5 and rBlo t 21, although in a few sera there was also a high level of IgE cross-reaction between the helminth extract and the recombinant allergens. Cross-reactivity to both recombinant allergens was observed in the same sera (data not shown). This finding could be explained if the anti-*Ascaris* antibody response in these sera donors had been more polyclonal than the immune response of the other serum donors, so that antibodies against additional cross-reactive epitopes would be produced. Indeed, it is known that the repertoire of the antigens that are recognized in complex antigenic mixtures may vary greatly in different individuals of the same species [34].

The observed difference in the degree of absorption by *A. lumbricoides* extract of anti-rBlo t 5 and anti-rBlo t 21 in relation to anti-BtE antibodies cannot be explained by the presence of lower levels of anti-recombinant allergen antibodies, since the amounts of anti-rBlo t 5 IgE antibody activity, on the contrary, were found to be higher than those of anti-BtE IgE antibody activity. Due to this less intense cross-reactivity, assays using a mixture of rBlot 5 and rBlo t 21 as antigen should be more specific than assays using the crude extract.

IgE antibodies raised by helminths can cross-react with several epitopes of mite antigenic extracts often used in diagnosis of allergic diseases possibly affecting the correct diagnosis. It becomes increasingly relevant, therefore, to obtain more specific allergens to make an accurate diagnosis of allergy and, consequently, improving immunotherapy for mite allergy. However, the price of assays using bacteria-produced recombinant antigens, as compared to those of assays using crude extracts obtained from mite cultures, is a factor that should be taken into consideration.

## Conclusions

We demonstrated that rBlo t 5 and rBlo t 21 were antigenic for the *B. tropicalis*-sensitized population evaluated in this work. In addition, we showed that the IgE reactive to these allergens had less cross-reactivity with *A. lumbricoides* extract than anti-*B. tropicalis* extract IgE, as assessed by an IgE-binding inhibition assay. Thus, rBlo t 5 and rBlo t 21 expressed in *E. coli* may be potential candidates to be used in a pool of different recombinant allergens for improving serodiagnosis assays of allergy to *B. tropicalis*. More studies are needed to obtain other recombinant allergens in order to develop a highly sensitive and specific assay for the diagnosis of allergy induced by *B. tropicalis* allergens.

## Abbreviations

rBlo t: *Blomia tropicalis* recombinant antigen; cDNA: Complementary DNA; pRSETA: Plasmidial vector pUC-derived expression vectors that designed for high-level protein expression and purification from cloned genes in *Escherichia coli*; BtE: *B. tropicalis* crude extract; ELISA: Enzyme-linked immunosorbent assay; SPT: Skin prick test; CEAR: Centro de Estudos em Alergias Respiratórias; SCAALA: Social Change in Asthma and Allergy in Latin America; mRNA: Messenger RNA; PCR: Polymerase chain reaction; BLAST: Basic Local Alignment Search Tool; IPTG: Isopropyl β-D-1-thiogalactopyranoside; SDS-PAGE: sodium dodecyl sulfate polyacrylamide gel electrophoresis; PVDF: Immobilon-P polyvinylidene difluoride; PBS: Phosphate-buffered saline; PBS/T: Phosphate-buffered saline containing 0.05% Tween; NFM: Non-fat milk; OD: Optical density; CONEP: National Commission on Ethics in Research.

## Competing interests

All authors report no conflict of interest financial or otherwise, with the findings of this study.

## Authors' contributions

KAC: carried out the laboratory assays and wrote the first draft of the manuscript; OPMN: helped in the study design, supervised the molecular biology work and helped in the manuscript revision; FBM: helped with the molecular biology assays; JCMP: helped in the inhibitory assays and revised the manuscript; FABF: helped in the imunmologial assay and revision the manuscript; GL, AAC and MLB provided the asthma patients and children sera and revised the manuscript; MCAS: has performed the statistical analysis; LCPC: helped in the study design and reviewed the manuscript; NMAN: conceived the work, the study design and supervised the immunological assays; CSP: helped in obtaining the Blo t 5 and Blo t 21. All authors read and approved the final manuscript.

## References

[B1] Bousquet J Dahl R Khaltaev N Global alliance against chronic respiratory diseases Allergy 2007 62 216 223 10.1111/j.1398-9995.2007.01307.x 17298337

[B2] Caraballo L Puerta L Martinez B Moreno L Identification of allergens from the mite Blomia tropicalis Clin Exp Allergy 1994 24 1056 1060 10.1111/j.1365-2222.1994.tb02743.x 7874604

[B3] Chua KY Cheong N Kuo IC Lee BW Yi FC Huang CH Liew LN The Blomia tropicalis allergens Protein Pept Lett 2007 14 325 333 10.2174/092986607780363862 17504089

[B4] Gao YF Wang De Y Ong TC Tay SL Yap KH Chew FT Identification and characterization of a novel allergen from Blomia tropicalis: Blo t 21 J Allergy Clin Immunol 2007 120 105 112 10.1016/j.jaci.2007.02.032 17445876

[B5] Tan KW Ong TC Gao YF Tiong YS Wong KN Chew FT Mok YK NMR Structure and IgE Epitopes of Blo t 21, a Major Dust Mite Allergen from Blomia tropicalis J Biol Chem 2012 287 34776 34785 10.1074/jbc.M112.348730 22887997 PMC3464580

[B6] Bhalla PL Singh MB Biotechnology-based allergy diagnosis and vaccination Trends Biotechnol 2008 26 153 161 10.1016/j.tibtech.2007.11.010 18222557

[B7] Smith HE Hogger C Lallemant C Crook D Frew AJ Is structured allergy history sufficient when assessing patients with asthma and rhinitis in general practice? J Allergy Clin Immunol 2009 123 646 650 10.1016/j.jaci.2008.11.005 19135237

[B8] Chan SL Ong TC Gao YF Tiong YS Wang De Y Chew FT Mok YK Nuclear magnetic resonance structure and IgE epitopes of Blo t 5, a major dust mite allergen J Immunol 2008 181 2586 2596 18684949 10.4049/jimmunol.181.4.2586

[B9] Ponte JC Junqueira SB Veiga RV Barreto ML Pontes-de-Carvalho LC Alcantara-Neves NM A study on the immunological basis of the dissociation between type I-hypersensitivity skin reactions to Blomia tropicalis antigens and serum anti-B. tropicalis IgE antibodies BMC Immunol 2011 12 34 10.1186/1471-2172-12-34 21631925 PMC3118201

[B10] Acevedo N Caraballo L IgE cross-reactivity between Ascaris lumbricoides and mite allergens: possible influences on allergic sensitization and asthma Parasite Immunol 2011 33 309 321 10.1111/j.1365-3024.2011.01288.x 21388422

[B11] Baqueiro T Russo M Silva VM Meirelles T Oliveira PR Gomes E Barboza R Cerqueira-Lima AT Figueiredo CA Pontes-de-Carvalho L Alcantara-Neves NM Respiratory allergy to Blomia tropicalis: immune response in four syngeneic mouse strains and assessment of a low allergen-dose, short-term experimental model Respir Res 2010 11 51 10.1186/1465-9921-11-51 20433763 PMC2890645

[B12] Lowry OH Rosebrough NJ Farr AL Randall RJ Protein measurement with the Folin phenol reagent J Biol Chem 1951 193 265 275 14907713

[B13] Laemmli UK Cleavage of structural proteins during the assembly of the head of bacteriophage T4 Nature 1970 227 680 685 10.1038/227680a0 5432063

[B14] Barreto ML Cunha SS Alcantara-Neves N Carvalho LP Cruz AA Stein RT Genser B Cooper PJ Rodrigues LC Risk factors and immunological pathways for asthma and other allergic diseases in children: background and methodology of a longitudinal study in a large urban center in Northeastern Brazil (Salvador-SCAALA study) BMC Pulm Med 2006 6 15 10.1186/1471-2466-6-15 16796729 PMC1559717

[B15] Chew FT Zhang L Ho TM Lee BW House dust mite fauna of tropical Singapore Clin Exp Allergy 1999 29 201 206 10.1046/j.1365-2222.1999.00493.x 10051724

[B16] Castro Almarales RL Mateo Morejon M Naranjo Robalino RM Navarro Viltre BI Alvarez Castello M Ronquillo Diaz M Garcia Gomez I Oliva Diaz Y Gonzalez Leon M Rodriguez Canosa JS Labrada Rosado A Correlation between skin tests to Dermatophagoides pteronyssinus, Dermatophagoides siboney and Blomia tropicalis in Cuban asthmatics Allergol Immunopathol (Madr) 2006 34 23 26 10.1157/13084223 16540067

[B17] Yu MK Lin CY Chen WL Chen CT Prevalence of Blomia tropicalis in wheezing children in central Taiwan J Microbiol Immunol Infect 2008 41 68 73 18327429

[B18] Baqueiro T Carvalho FM Rios CF dos Santos NM Alcantara-Neves NM Dust mite species and allergen concentrations in beds of individuals belonging to different urban socioeconomic groups in Brazil J Asthma 2006 43 101 105 10.1080/02770900500497958 16517425

[B19] Kidon MI Chiang WC Liew WK Ong TC Tiong YS Wong KN Angus AC Ong ST Gao YF Reginald K Mite component-specific IgE repertoire and phenotypes of allergic disease in childhood: the tropical perspective Pediatr Allergy Immunol 2011 22 202 210 10.1111/j.1399-3038.2010.01094.x 21332797

[B20] Valenta R Lidholm J Niederberger V Hayek B Kraft D Gronlund H The recombinant allergen-based concept of component-resolved diagnostics and immunotherapy (CRD and CRIT) Clin Exp Allergy 1999 29 896 904 10.1046/j.1365-2222.1999.00653.x 10383589

[B21] Weghofer M Thomas WR Kronqvist M Mari A Purohit A Pauli G Horak F Gronlund H van Hage M Valenta R Vrtala S Variability of IgE reactivity profiles among European mite allergic patients Eur J Clin Investig 2008 38 959 965 10.1111/j.1365-2362.2008.02048.x 19021722

[B22] Yi FC Chua KY Cheong N Shek LP Lee BW Immunoglobulin E reactivity of native Blo t 5, a major allergen of Blomia tropicalis Clin Exp Allergy 2004 34 1762 1767 10.1111/j.1365-2222.2004.02107.x 15544602

[B23] Valmonte GR Cauyan GA Ramos JD IgE cross-reactivity between house dust mite allergens and Ascaris lumbricoides antigens Asia Pacific Allergy 2012 2 35 44 10.5415/apallergy.2012.2.1.35 22348205 PMC3269600

[B24] Erwin EA Platts-Mills TA Allergens Immunol Allergy Clin North Am 2005 25 1 14 10.1016/j.iac.2004.09.008 15579361

[B25] Taketomi EA Almeida KP Silva DAO FL Allergens: sources, exposure and sensitization levels, diagnostic tools and immunotherapeutical applications J Med Med Sci 2010 1 580 588

[B26] Vrtala S From allergen genes to new forms of allergy diagnosis and treatment Allergy 2008 63 299 309 10.1111/j.1398-9995.2007.01609.x 18269675

[B27] van Ree R Analytic aspects of the standardization of allergenic extracts Allergy 1997 52 795 805 10.1111/j.1398-9995.1997.tb02150.x 9284978

[B28] Chapman MD Smith AM Vailes LD Arruda LK Dhanaraj V Pomes A Recombinant allergens for diagnosis and therapy of allergic disease J Allergy Clin Immunol 2000 106 409 418 10.1067/mai.2000.109832 10984358

[B29] Cooper PJ Chico ME Bland M Griffin GE Nutman TB Allergic symptoms, atopy, and geohelminth infections in a rural area of Ecuador Am J Respir Crit Care Med 2003 168 313 317 10.1164/rccm.200211-1320OC 12714349

[B30] Alcantara-Neves NM Badaro SJ dos Santos MC Pontes-de-Carvalho L Barreto ML The presence of serum anti-Ascaris lumbricoides IgE antibodies and of Trichuris trichiura infection are risk factors for wheezing and/or atopy in preschool-aged Brazilian children Respir Res 2010 11 114 10.1186/1465-9921-11-114 20731833 PMC2939601

[B31] Dattoli VC Freire SM Mendonca LR Santos PC Meyer R Alcantara-Neves NM Toxocara canis infection is associated with eosinophilia and total IgE in blood donors from a large Brazilian centre Trop Med Int Health 2011 16 514 517 10.1111/j.1365-3156.2010.02719.x 21410848

[B32] Rodrigues LC Newcombe PJ Cunha SS Alcantara-Neves NM Genser B Cruz AA Simoes SM Fiaccone R Amorim L Cooper PJ Barreto ML Early infection with Trichuris trichiura and allergen skin test reactivity in later childhood Clin Exp Allergy 2008 38 1769 1777 18547322 10.1111/j.1365-2222.2008.03027.x

[B33] Acevedo N Sanchez J Erler A Mercado D Briza P Kennedy M Fernandez A Gutierrez M Chua KY Cheong N IgE cross-reactivity between Ascaris and domestic mite allergens: the role of tropomyosin and the nematode polyprotein ABA-1 Allergy 2009 64 1635 1643 10.1111/j.1398-9995.2009.02084.x 19624559

[B34] Teixeira MC Oliveira GG Silvany MA Alcantara-Neves NM Soares MB Ribeiro-Dos-Santos R Jeronimo SM Costa CH Dos-Santos WL Eichinger D Pontes-de-Carvalho L A strategy for identifying serodiagnostically relevant antigens of Leishmania or other pathogens in genetic libraries Biologicals 2007 35 51 54 10.1016/j.biologicals.2006.01.005 16580229

